# Berberine Suppression of Human IgE but Not IgG Production via Inhibition of STAT6 Binding Activity at IgE Promoter by BCL6

**DOI:** 10.3390/cells14080591

**Published:** 2025-04-14

**Authors:** Anish R. Maskey, Michelle Carnazza, Madison Spears, Steven Hemmindinger, Daniel Kopulos, Nan Yang, Humayun K. Islam, Augustine L. Moscatello, Jan Geliebter, Raj K. Tiwari, Xiu-Min Li

**Affiliations:** 1 Department of Pathology, Microbiology & Immunology, New York Medical College, Valhalla, NY 10595, USA; amaskey@nymc.edu (A.R.M.); mspears@student.nymc.edu (M.S.); dkopulos@student.nymc.edu (D.K.); humayun_islam@nymc.edu (H.K.I.); jan_geliebter@nymc.edu (J.G.); raj_tiwari@nymc.edu (R.K.T.); 2 Division of R&D, General Nutraceutical Technology LLC., Elmsford, NY 10523, USA; michelle.carnazza@gnt-us.com (M.C.); nan.yang@gnt-us.com (N.Y.); 3 Department of Otorhinolaryngology, Westchester Medical Center, Valhalla, NY 10595, USA; steven.hemmerdinger@wmchealth.org (S.H.); augustine.moscatello@wmchealth.org (A.L.M.); 4 Department of Pathology, Westchester Medical Center, Valhalla, NY 10595, USA; 5 Department of Dermatology, New York Medical College, Valhalla, NY 10595, USA

**Keywords:** IgE, anti-CD40/IL4, tonsils, STAT6, BCL6

## Abstract

IgE may lead to life-threatening anaphylaxis. Currently, no satisfactory treatment to inhibit IgE production exists. This study aims to explore the anti-IgE effect of berberine (BBR) and possible mechanisms using human tonsil cells. Tonsil cells were treated with BBR at different doses following stimulation with anti-CD40/IL4 alone or in combination with poly I:C and Pam3CSK4 for 10 or 4 days. IgE and IgG levels were determined by ELISA and cell viability by trypan blue exclusion. Gene expression was analyzed by qRT-PCR and affinity binding assay was performed by chromatin immunoprecipitation assay (ChIP). BBR showed dose-dependent inhibition of IgE production following anti-CD40/IL4 stimulation without affecting cell viability and IgG levels. BBR (10 µg/mL) completely inhibited IgE production by B cells stimulated with anti-CD40/IL4 in combination with vaccine adjuvants—poly I:C and Pam3CSK4 without affecting IgG levels and cell viability. BBR inhibited IgE heavy chain (IgEh), epsilon germline-transcript (εGLT), STAT6, and NFκB1 and enhanced IFN-γ, NFκB1A, and BCL6 gene expression. ChIP assay showed that BBR inhibited STAT6 binding in the IgEh promoter region by enhancing BCL6 binding. This study shows BBR regulates IgE in human tonsil cells by inhibiting STAT6 binding through BCL6 at the IgEh promoter showing its potential for treating IgE-mediated allergies.

## 1. Introduction

Allergic disease is a growing health concern as its prevalence has increased dramatically over the last several decades, affecting over 25% of adults and reaching almost 50% of children worldwide [1]. It is the result of a specific and reproducible immune response occurring upon exposure to an allergen. Symptoms manifest where confrontation occurs due to the release of histamine and other inflammatory mediators. Activation of these mediators for a Type 1 hypersensitivity reaction is owed to the production of immunoglobulin E (IgE), which can lead to life-threatening reactions, including anaphylaxis. IgE is produced as a result of food sensitization to specific food antigens and airborne allergens, a result of a Th2-cytokine milieu high in IL-4, IL-5, and IL-13 [2]. The production of IgE requires engagement with anti-CD40/IL-4. This interaction enhances germline Cε transcription and subsequently induces isotype switching to IgE [3,4]. IgE binds at high affinity to FcεRI receptors present in cells, including mast cells, eosinophils, and basophils. Activation of these cells results in their degranulation of enzymes, toxic mediators, lipid mediators, and cytokines, which cause symptoms such as tingling or oropharyngeal itching, hives, swelling, wheezing, vomiting, diarrhea, and abdominal pain, or, more severely, life-threatening anaphylaxis [5].

Cytokine-driven B cell class switch recombination (CSR) is critical in allergic responses, enabling the transition from IgM to IgE production, which further drives Type 1 hypersensitivity reactions. Cytokines IL-4 and IL-13 bind to their cognate B cells receptors, which induce activation of the transcription factor signal transducer and activator of transcription 6 (STAT6) [6]. Subsequently, STAT6 can bind to its binding site on the IgE promoter, and the transcription of IgE then occurs [6]. While STAT6 is the most critical inducer of IgE, there are additional binding sites in the promoter region of IgE for nuclear factor kappa B (NFκB), paired box 5 (Pax5), and CCAAT-enhancer-binding proteins (C/EBP) [6]. (B-cell lymphoma 6) BCL6 is a transcription factor primarily known for its role in germinal center (GC) B cell formation and regulation of B cell differentiation. BCL6 acts as a negative regulator of IgE CSR. When BCL6 expression is high, B cells are less likely to undergo IL-4–induced IgE switching, preventing excessive IgE production [7]. Interestingly, BCL6 has demonstrated negative regulatory abilities, binding to the STAT6-binding site and, therefore, repressing induction of IgE transcription [6]. Similarly, an inhibitor of nuclear factor kappa B (IκB) inhibits NFκB, preventing binding at the IgE promoter and, consequently, its transcription. Therefore, altering these signaling processes may be a more effective therapeutic strategy for allergic disease through preventing IgE production. This is advantageous over current strategies of avoidance, symptom alleviation, and sequestering of IgE after its production.

Currently, allergen immunotherapy is widely used to manage food allergies; however, it is associated with the risk of anaphylaxis and the need for long-term treatment. Other approaches include the use of corticosteroids and antihistamines, which focus on improving symptoms and are associated with negative side effects and a high risk of relapses following cessation of therapy. Therefore, a great therapeutic barrier to overcome is the inability to prevent IgE from being produced. Efforts to decrease IgE levels in food allergies are widely explored. An anti-IgE drug Omalizumab, has shown promising results to treat IgE-mediated allergies. However, its use has often been associated with side effects ranging from local skin inflammation at the injection site to systemic anaphylaxis [8]. Additionally, Omalizumab binds circulating IgE but does not directly suppress B-cell IgE production. Similarly, natural formula-Food allergy herbal formula-2 (FAFH2) derived from its original 10-herb formula FAFH1 by eliminating two herbs-Xi-Xin and Zhi-Fu-Zi, to enhance the protective effect and safety profile of the formula has shown promising effects [9]. FAHF2 and its purified form, B-FAHF2, have previously been shown to lower peanut-specific IgE and prevent peanut-induced anaphylaxis in mice models [10,11]. Furthermore, it has been shown to decrease IgE production in in-vitro B cells and in peripheral blood mononuclear cells (PBMCs) isolated from peanut-allergic patients [12]. Based on these results, natural compound-based therapies are widely studied in IgE-mediated allergies.

Naturally occurring compounds have demonstrated immunomodulatory properties and, therefore, have been investigated in the context of allergic disease. Studies primarily include in vitro, in vivo, and ex vivo analysis. Investigation into the active components of natural compounds responsible for the alleviation of IgE-mediated responses has been highlighted. Berberine (BBR), an isoquinoline compound, has previously been shown to inhibit IgE production in vitro [13] and in human PBMCs following anti-CD40/IL4 stimulation [12] and to reduce mast cell degranulation in a dose-dependent manner (Maskey et al., 2025, manuscript in preparation). Furthermore, berberine-containing natural medicine combined with boiled peanut oral immunotherapy showed a sustained reduction of IgE and a number of IgE^+^ B cells [14]. Mechanistically, BBR has been shown to reduce the expression of transcription factors x-box binding protein (Xbp1), phosphorylated IκBα, and STAT6 while increasing the expression of signal transducer and activator of transcription 1 (STAT1), and T-box transcription factor (Tbet) [12,13]. Human tonsil samples offer a valuable model for investigating allergen-specific immune responses due to their abundant B and T cell populations. This study explores the anti-IgE effects of the natural compound berberine (BBR) and seeks to elucidate the underlying mechanisms following anti-CD40/IL-4 stimulation. By isolating single cells from fresh human tonsils obtained during tonsillectomy, we aim to further our understanding of the molecular pathways involved in IgE inhibition, thereby informing potential therapeutic strategies for allergic diseases.

## 2. Materials and Methods

### 2.1. Tonsil Collection

Children (4–8 yrs.) undergoing routine tonsillectomy (*n* = 10; 5 male and 5 female) at Westchester Medical Center (WMC) consented, following New York Medical College Institutional Review Board (NYMC IRB) approval protocol (IRB #12874). Out of 10 patients, 3 were clinically diagnosed with obstructive sleep apnea (OSA), 4 were reported to have enlarged tonsils, 2 had tonsillitis, and 1 had tonsil hypertrophy (Table 1). None of these patients had any forms of allergies. Freshly removed tonsils were immediately stored in ice-cold RPMI 1640 and transported to the lab.

### 2.2. Tonsil Processing and Single Cell Isolation

Tonsils were washed with cold PBS twice. Single-cell suspension was prepared from the tissue as previously described [15,16]. Briefly, tissue was fragmented mechanically using a sterilized blade into 3–10 mm pieces and passed through a 100 µm cell strainer, ensuring all the fragments passed through in a tube containing cold RPMI 1640. This cell suspension was overlayed onto a Ficoll with minimum agitation and without mixing. The mixture was centrifuged at 7000 rpm with no break for 20 min at room temperature. The fluffy white layer containing T and B cells at the interface between the Ficoll and the medium was collected using a pipette. The cell pellet was washed three times with ice-cold PBS and re-suspended on ice-cold RPMI 1640. The cell count was performed using a hemocytometer. A variable number of cells was obtained from each tonsil based on size of the tissue removed during surgery. Depending on the number of cells obtained, experiments were carried out for respective assays used in this manuscript.

### 2.3. High-Performance Liquid Chromatography (HPLC) Analysis of Berberine

Berberine compound tested in this study was obtained from Xi’an SaiYang Biotechnology, LLC (Xi’an, China) and analyzed using high-performance liquid chromatography (HPLC). Moreover, 10 µL of BBR solution (400 µg/mL) was injected into a Waters 2690 separation module coupled with a 2996 PDA detector (Waters, Milford, MA, USA) and separated on a ZORBAX SB-C18 (4.6 × 150 mm, 5 µm) column (Agilent, Santa Clara, CA, USA). The mobile phase used was 0.1% formic acid in water solution (mobile phase A) and HPLC grade acetonitrile with 0.1% formic acid (Fisher Scientific, Fair Lawn, NJ, USA, mobile phase B). The flow rate was set as 1 mL/min. The linear gradient separation was achieved as follows: 10% mobile phase B to 100% over 10 min and maintained at 100% mobile phase B for 3 min. The chromatographic data were collected at wavelength 264 nm and processed using Waters Empower 3 software (Figure S1).

### 2.4. Cell Stimulation

Cell concentration was adjusted to 4 × 10^6^/mL in RPMI 1640 containing 10% Heat-inactivated FBS and 1% penicillin/streptomycin antibiotic cocktail. Cells were stimulated with human anti-CD40 (1 µg/mL) (Fisher Scientific, Pittsburgh, PA, USA) and recombinant human IL-4 (100 ng/mL) (R&D systems, Minneapolis, MN, USA). BBR was added at different doses (0.6, 1.2, 2.5, 5, 10, 20 µg/mL) to respective culture conditions. Cells were incubated for 10 days in 37 degrees incubator supplied with 5% CO_2_. In a separate experiment, cells were stimulated with anti-CD40/IL-4 in combination with Toll-like receptor (TLR) agonists-poly I:C (TLR3), Pam3CSK4 (TLR2), and both. BBR treatment was added to the culture at 10 µg/mL. The culture was incubated at 37 degrees for 4 days. Cell supernatant was harvested, IgE and IgG levels were determined by ELISA (Mabtech, Cincinnati, OH, USA) following manufacturer’s protocol, and the cell pellet was subjected to RNA extraction for gene expression analysis.

### 2.5. Cell Viability

Cell viability was evaluated by trypan blue excursion. Briefly, 10 µL of cells were mixed with equal volume of trypan blue and loaded onto a hemocytometer, and cells were counted under a microscope. The percentage of viable cells was calculated as viable cells (%) = (total number of viable cells)/(total number of cells) × 100.

### 2.6. Antibody Measurement

IgE and IgG levels were measured by ELISA (Mabtech, Cincinnati, OH, USA) following manufacturer’s protocol. Briefly 96-well plate was coated with respective capture antibodies and incubated overnight at 4 degrees. Samples were added to each well and incubated for 2 h at room temperature. Biotinylated detection antibody followed by streptavidin-HPR was added to each well. TMB substrate was added to develop color reaction for 15 min, and the absorbance was finally measured following stoppage of reaction by adding 0.2 M H_2_SO_4_. The optical density was read at 450 nm, and the values were quantified using the standard curve.

### 2.7. Quantitative Real-Time PCR

Cell pellets were used for RNA extraction, as previously described [17]. Briefly, RNA was extracted by homogenizing the cells in RLT buffer and using the Qiagen mini kit. For each qRT-PCR, a 25 µL reaction was run with 12.5 µL Maxima SYBR Green/ROX qPCR Master Mix 2X, 1.8 µL of 0.3 µM assays on demand primer, and 300 ng of RNA. The qRT-PCR was performed at 40 cycles of 25 °C for 5 min, 42 °C for 60 min, and 70 °C for 15 min. The Ct values for each gene were normalized by subtracting the Ct values for the housekeeping gene GAPDH (ΔCT). The relative fold change in mRNA expression between groups was calculated and expressed as 2^−ΔΔCT^. The primer sequences used are shown in Table S1.

### 2.8. The Chromatin Immunoprecipitation (ChIP) Assay

ChIP assay was performed with the SimpleChIP^®^ Plus Enzymatic Chromatin IP Kit (Cell Signaling Technology, Danvers, MA, USA), following manufacturer’s instructions. Briefly, cells were fixed and crosslinked by incubation with formaldehyde followed by glycine and washed by centrifugation with ice-cold PBS. Nuclei were prepared in the presence of the buffers provided by the kit and DTT. Micrococcal nuclease was added, and digestion was stopped with the addition of 0.5 M EDTA. Nuclei were pelleted and resuspended in ChIP buffer for sonication. Following sonication and centrifugation, the supernatant was collected, and 50 µL was used for analysis of chromatin digestion and concentration utilizing the provided RNAse A and Proteinase K. After DNA purification, DNA fragment size, and concentration were determined by 1% agarose gel electrophoresis and OD_260_. 10 µg of digested chromatin preparation was used for chromatin immunoprecipitation. Following resuspension in the ChIP buffer, the digested, cross-linked chromatin preparation was added to the immunoprecipitating antibody at a concentration indicated by the ChIP-compatible CST antibody, including the positive and negative controls provided. The samples were then resuspended in the provided magnetic beads and pelleted by placing them on a magnetic separation rack. This was immediately followed by a low salt wash and a high salt wash. Elution of the chromatin from the antibody/magnetic beads was performed in the presence of the elution buffer provided in the kit at 65 °C with gentle vortexing. Reversal of crosslinking was performed through the addition of the provided 5 M NaCl and Proteinase K. DNA purification was performed again with the provided purification kit. Real-time quantitative PCR was performed with qPCR master mix (qPCRBio) and 10 µM of our primers on the QuantStudio 5 Real-Time PCR Instrument (96-Well) (ThermoFisher Scientific, IL, USA). Initial denaturation at 95 °C for 3 min followed by 40 cycles of denaturation at 95 °C for 15 s and then anneal and extension at 60 °C for 60 s. The following primer sequence was used for ChIp assay, *BCL6* (f-CTGGCTTTTGTGACGGAAAT; r-AACCTGAAAACCCACACTCG) and STAT6 (f-GGCAGGGAATGGTAGTGGATAG; r-TCAATCAGGGCCTCACCGTA).

### 2.9. Statistical Analysis

For normally distributed data, comparisons between two independent groups were performed using one-way analysis of variance (ANOVA) followed by Bonferroni correction. For skewed data, non-parametric statistical method was applied, and comparisons across multiple independent groups were performed using the Kruskal–Wallis test. When significant differences were observed, post hoc pairwise comparisons were conducted using Dunn’s test. A *p* value less than or equal to 0.05 was considered statistically significant. All statistical analyses were performed using GraphPad Prism (version 10.0).

## 3. Results

### 3.1. Berberine Dose Dependently Inhibited IgE Production Following Anti-CD40/IL-4 Stimulation

**Figure 1:**
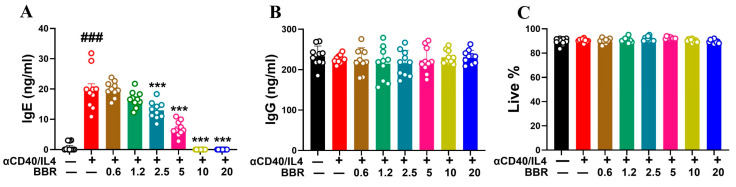
Berberine suppressed IgE production by tonsil cells following T-cell-dependent stimulation without any effect on IgG. Tonsil cells were obtained from patients undergoing tonsillectomy. Single-cell suspensions were prepared and stimulated T-cell-dependent co-stimulation provided by anti-CD40/IL4. BBR was added to the culture at different doses (0.6, 1.2, 2.5, 5, 10, 20 µg/mL). Cells were cultured for 10 days, and IgE and IgG levels were measured. (**A**) BBR suppressed IgE production dose dependently. (**B**) BBR showed no effect on IgG production. (**C**) BBR showed no effect on cell viability. Different colors indicate different doses of BBR. Data represents mean ± SD; *n* = 10 tonsils; ^###^ *p* < 0.01 vs. unstimulated; *** *p* < 0.001 vs. stimulated/untreated; Data was analyzed using one-way ANOVA.

CD4^+^ T cells provide co-stimulation through CD40-CD40L interaction and in the presence of IL-4 cytokine to induce class switching of B cells. In the presence of anti-CD40/IL-4, there was a robust increase in IgE production by tonsil cells (*p* < 0.001). BBR treatment showed a dose-dependent inhibition of IgE production by tonsil cells with 10 µg/mL and 20 µg/mL showing > 95% inhibition (*p* < 0.001) (Figure 1A). Furthermore, in order to validate BBR’s specific anti-IgE effect, we measured the levels of IgG and demonstrated that BBR did not have any significant effect on IgG levels (Figure 1B). BBR treatment showed 100% cell viability as compared to untreated control (Figure 1C). Overall, these results highlight BBR’s anti-IgE effects without any cell cytotoxicity and effect on IgG.

### 3.2. Berberine Suppressed IgE Production by Tonsil Cells Following T-Cell-Dependent Stimulation with Poly IC and Pam3CSK4

**Figure 2:**
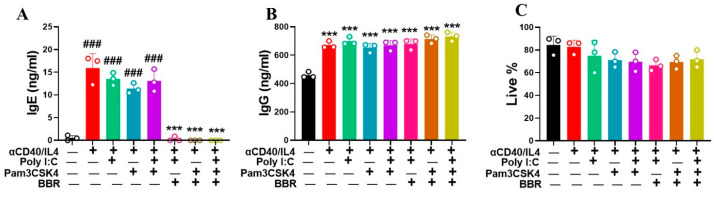
Berberine suppressed IgE production by tonsil cells following T-cell-dependent stimulation with poly IC and Pam3CSK4 without any effect on IgG. Tonsil cells were obtained from patients undergoing tonsillectomy. Single-cell suspensions were prepared and stimulated with poly I:C (25 μg/mL), Pam3CSK4 (1 μg/mL), or a combination of both with T-cell-dependent co-stimulation provided by anti-CD40/IL4. BBR was added to the culture at 10 µg/mL. Cells were cultured for 4 days to measure IgE and IgG levels. (**A**) BBR suppressed IgE production. (**B**) BBR showed no effect on IgG production. (**C**) BBR showed no effect on cell viability. Color bars indicate different culture conditions. Data represents mean ± SD; *n* = 3 tonsils, triplicate culture; ^###^ *p* < 0.01 vs. unstimulated; *** *p* < 0.001 vs. stimulated/untreated. Data were analyzed using one-way ANOVA.

TLR agonists are widely studied and may contribute to enhanced cell activation through the secretion of cytokines and proliferation. We used two TLR agonists—Poly: IC and Pam3CSK4 to study their effect on IgE and IgG production following BBR treatment. Interestingly, our results showed that using Poly: IC and Pam3CSK4 in combination with CD40/IL4 had a significant effect on IgE production by tonsil cells (Figure 2A; *p* < 0.001). With BBR (10 µg/mL), there was complete inhibition of IgE production using the same TLR combination stimulation strategy (Figure 2A, *p* < 0.001). Furthermore, none of the TLR agonists, either alone or in combination with CD40/IL4, showed any cell cytotoxicity (Figure 2C). These results indicate that BBR potentially has a great role in inhibiting IgE production independent of stimulus.

### 3.3. Berberine Inhibited IgE Heavy Chain and Epsilon GLT mRNA Expression

**Figure 3:**
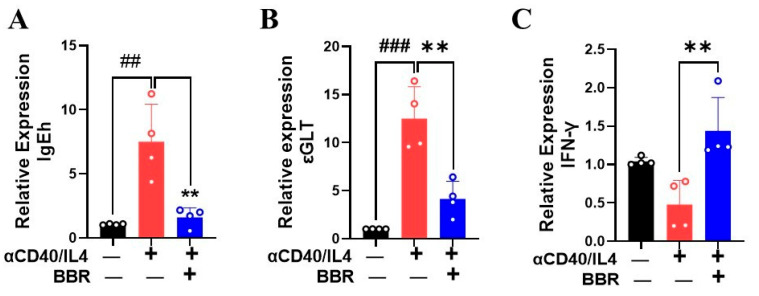
Berberine inhibited IgEh and εGLT and increased IFNγ by tonsil cells in response to antiCD40/IL-4 stimulation. Tonsil cells were obtained from patients undergoing tonsillectomy. Single-cell suspensions were prepared and stimulated with anti-CD40/IL4. BBR was added to the culture at 10 µg/mL. Cells were cultured for 4 days, and cell pellets were used for RNA extraction and gene expression analysis by qRT-PCR. Berberine treatment inhibited the expression levels of IgEh (**A**) and εGLT (**B**) and increased the expression levels of IFN-γ (**C**). Color bars indicate different culture conditions. Data represent mean ± SD; *n* = 4. ^##^ *p* < 0.01; ^###^ *p* < 0.001 vs. unstimulated; ** *p* < 0.01 vs. BBR. Data was analyzed using one-way ANOVA.

IgE heavy chain (IgEh) and εGLT play a critical role in IgE class switching. Previously, BBR has been shown to inhibit εGLT by human PBMC [12]. To investigate the effect of BBR on IgEh and εGLT following anti-CD40/IL4 stimulation using human tonsils, mRNA expression was measured. We showed that BBR at 10 µg/mL significantly decreased the expression of IgEh and εGLT as compared to the stimulated control (Figure 3A,B; *p* < 0.01). Additionally, IFN-γ has been reported previously to inhibit IgE-mediated degranulation of mast cells [18], suppress immunoglobulin IgE class switching [19], and induce IgG production [20]. Moreover, the Th1 response and cytokine IFN-γ counterbalances the Th2 response by inhibiting the production of IL-4 and IgE [21]. Our results showed that BBR increased the expression of IFN-γ in single cells isolated from human tonsils following anti-CD40/IL4 stimulation (Figure 3C; *p* < 0.01). Overall, these results indicate that BBR has the ability to inhibit IgE class switching by inhibiting the expression of IgEh and εGLT while increasing the expression of IFN-γ.

### 3.4. Berberine Increased BCL6 and Decreased STAT6 Expression

**Figure 4:**
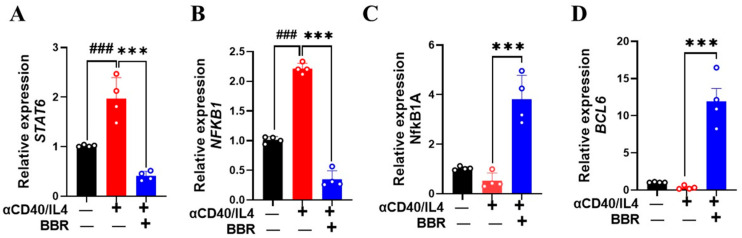
Berberine modulated genes associated with IgE regulation. Tonsil cells were obtained from patients undergoing tonsillectomy. Single-cell suspension was prepared and stimulated with anti-CD40/IL4. BBR was added to the culture at 10 µg/mL. Cells were cultured for 4 days, and cell pellets were used for gene expression analysis by qRT-PCR. Berberine treatment decreased expression levels of STAT6 (**A**) and NFKB1 (**B**) and increased expression levels of NFkB1A (**C**) and BCL6 (**D**). Color bars indicate different culture conditions. Data represent mean± SD *n* = 4. ^###^ *p* < 0.01 vs. unstimulated; *** *p* < 0.01 vs. stimulated but untreated cells. Data were analyzed using one-way ANOVA.

BCL6 is a sequence-specific transcriptional repressor expressed in germinal B cells and is known to suppress the expression of Cε germline transcript [22]. The binding of IL-4 on its B cell receptors leads to the activation of transcription factor STAT6 and the induction of germline transcription at the Cε locus [6]. To explore BBR’s effect on tonsil B cells, we performed RT-PCR to analyze gene expression levels of BCL6 and STAT6. Cells treated with BBR (10 µg/mL) showed a significant decrease in STAT6 expression (Figure 4A; *p* < 0.001). Furthermore, the germline Cε promoter region also contains the binding site for nuclear factor-κB (NF-κB) [23], and stimulation with anti-CD40/IL-4 leads to NF-κB activation, promoting IgE production. Our results showed a significant decrease in NF-κB1 expression following BBR treatment as compared to anti-CD40/IL-4 stimulation alone (Figure 4B; *p* < 0.001). In addition, NFκB1A, the gene encoding the alpha subunit of the Iκκ complex, was significantly upregulated with BBR treatment (Figure 4C, *p* < 0.001). When NF-κB is inactive, it remains bound to the Iκκ complex, and specific signals trigger the activation of Iκκ complex, leading to the release of NF-κB. Similarly, BBR (10 µg/mL) showed a robust increase in BCL6 expression (Figure 4D; *p* < 0.01). Although STAT1 is an important signaling molecule for B-cell activation and functional regulation [24], its direct role in IgE production in the context of allergic response remains unknown.

### 3.5. Berberine Inhibited STAT6 Binding Activity but Increased BCL6 Binding Activity on IgE Promoter

**Figure 5:**
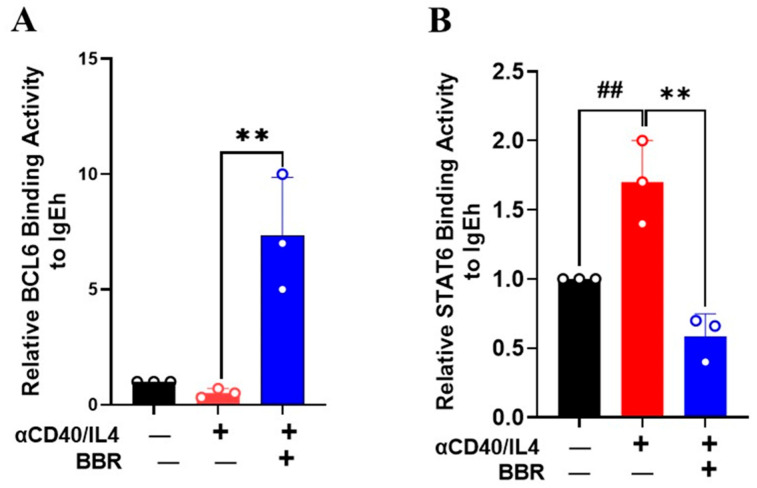
Berberine inhibited STAT6 binding on IgEh promoter. Tonsil cells were cultured with BBR (10 µg/mL) and stimulated with anti-CD40/IL-4 for 4 days. Cells were subjected to chromatin immunoprecipitation (ChIP) assay to determine relative binding activity of (**A**) Bcl6 and (**B**) STAT-6 on IgEh by qPCR. Color bars indicate different culture conditions. Data represent mean ± SD; *n* = 3. ^##^ *p* < 0.01 vs. unstimulated; ** *p* < 0.01 vs. BBR. Data were analyzed using one-way ANOVA.

To investigate the effect of BBR on STAT6 and BCL6 binding at the IgEh promoter, we performed a ChIP assay. We found that BCL6 binding was significantly enriched at the IgEh promoter upon BBR treatment (Figure 5A, *p* < 0.01). Additionally, we found that CD40/IL-4 treatment increased STAT6 enrichment at the IgE promoter (Figure 5B, *p* < 0.01); however, this enrichment was significantly reduced with BBR treatment (Figure 5B, *p* < 0.01). Overall, these findings suggest that BBR regulates IgEh transcription by modulating the binding of this transcription, which may be a potential mechanism for IgE inhibition.

## 4. Discussion

Tonsils are crucial components of the immune system as it has a diverse population of cells including B cells, T cells, macrophages, dendritic cells, and stromal cells. It is documented that B cells are the principal cellular components of tonsils, comprising between 50–90% of lymphocytes [25]. Here, we evaluated the IgE response from single-cell suspension from tonsils as these cells better reflect the response of human B cells in secondary lymphoid tissue. Fresh tonsil cells were used to stimulate with anti-CD40/IL-4 to assist with the class switching of B cells to produce IgE. Children presented to the Department of Otorhinolaryngology at WMC were diagnosed with either OSA, enlarged tonsils, tonsil hypertrophy, or tonsillitis requiring surgical removal of tonsils by tonsillectomy. In this study, we showed the anti-IgE effects of natural small molecule compound BBR using tonsil cells. Previously, BBR has been shown to inhibit IgE production in human PBMC from food-allergic patients [12] and in IgE-producing U266 cells [13]. Additionally, BBR-containing natural medicine, when combined with a boiled peanut oral immunotherapy, resulted in a sustained reduction of IgE as well as IgE+ B cells in a murine model of peanut allergy [14]. This study adds to previous research by showing the remarkable anti-IgE effect of BBR using human tonsils. In our study, we found a dose-dependent inhibition of IgE production by tonsils cells following stimulation with anti-CD40/IL-4. Furthermore, we showed that BBR’s effect was IgE-specific and had no effect on the ability of tonsil cells to produce total IgG. In addition, we showed no change in live cell count associated with BBR across all experimental groups. These results suggest that BBR has no effect on cell viability and has a specific anti-IgE effect without altering total IgG levels. Further studies with enriched B cells from tonsils should be performed to determine the effect of BBR on cellular and molecular levels.

Several TLR agonists have studied their role in enhancing the initiation of immune responses. TLRs are also expressed in B-cells, and upon activation, they have the ability to differentiate into antibody-producing plasma cells [26]. There is limited information on how B cells stimulated with TLR ligands enhance immune response. However, a previous study showed that B cells express different TLRs (TLR1–9) and function to activate in different ways by producing IFN-γ, IL-10, and IL-6 [27]. Furthermore, the combination of poly I:C and Pam3CSK4 has been reported to synergistically enhance the activation of B cells in vitro [28]. Additionally, using poly I:C (TLR3) and Pam3CSK4 (TLR1/2) adjuvant combination for influenza and anthrax vaccine enhanced antibody production by B-cell in vivo [28]. Here, we tested the anti-IgE effect of BBR following stimulation with anti-CD40/IL4 in combination with different TLR agonists—Poly I:C, PamCSK4, or both. Our results showed complete inhibition of IgE production by BBR in the presence of TLR agonists. These anti-IgE effects were not associated with any change in IgG levels and showed no evidence of cell cytotoxicity. Additionally, there was a robust increase in IgG response following stimulation of cells with poly I:C, Pam3CSK4 or both in combination with anti-CD40/IL4. These findings indicate that BBR exerts a specific anti-IgE effect on tonsil cells when stimulated with anti-CD40/IL4 in combination with TLR agonist, poly I:C, Pam3CSK4 or both. Future studies using B cells isolated from immunized mice needs to be considered to study the effect of BBR in vivo.

IgE production requires B cell activation and class switch recombination [29,30]. Class switch recombination involves the activation of the IgE ε-germline transcript. The presence of the IgEh transcript provides evidence of class switch recombination and ε-germline transcript activation. In our study, using anti-CD40/IL4 stimulation, we showed a significant increase in the expression of IgEh, indicating activation of -germline and class switching to IgE production. To study if BBR’s anti-IgE effect is associated with regulating the expression of IgEh, we performed qRT-PCR and showed that BBR significantly inhibited the expression of IgEh. This suggests that BBR has the ability to inhibit class switch recombination. Additionally, BBR has previously been shown to inhibit ε-GLT following anti-CD40/IL4 stimulation in human PBMC [12]. Our results were consistent with previous findings as we showed significant inhibition of ε-GLT transcript following BBR treatment in tonsil cells. Furthermore, our results show a significant increase in the expression of IFN-γ. IFN-γ has the ability to inhibit immunoglobulin class switching to IgE while enhancing IgG production [20]. These results suggest that BBR inhibition of IgE is at least in part due to an increase in the Th1 cytokine IFN-γ, which counterbalances the Th2 immune response in allergic diseases.

The binding of IL-4 to the B cell receptor leads to the activation of transcription factor STAT6 and the subsequent induction of germline transcript in the C ε locus [6]. The human ε-germline promoter region consists of several binding sites for transcription factors, including STAT6, BCL6, and NFκB. The presence of NFκB and STAT6 is essential for activating Ig Cε-germline transcription, which is necessary for class switching to IgE production [31]. Studies indicate that STAT6 and NFkB are closely positioned with each other within the Cε-germline promoter region, providing evidence that these interact directly in response to IL-4 stimulation [32,33]. As STAT6 plays an important role in IgE class switching, we measured the expression of STAT6 and showed BBR significantly reduced STAT6 expression following anti-CD40/IL4 stimulation. NFκB is a heterodimer complex containing REL-like domain proteins RELA/p65, RELB, NFκB1/p50, REL, and NFκB2/p52NFκB. NFκB complex proteins are held in the cytoplasm in an inactive state and are tightly controlled by the IκB subunit, inhibiting nuclear uptake of NFκB complex [34]. NFκB1A is the alpha subunit of the IκB complex and helps NFκB bind to the complex. When the NFκB1A protein receives an activation signal, it breaks down the inhibitory complex, allowing NFκB to translocate to the nuclease and bind to DNA. We showed a significant reduction in the expression of NFκB1 and increased expression in its inhibitor NFκB1A, highlighting the role of BBR in regulating transcription factors in antibody class switching to produce IgE. Although these results with a specific focus on a few highly relevant genes show the promising effect of BBR, single-cell RNA sequencing (scRNA-seq) of tonsil cells should be considered as it allows for a detailed examination of gene expression in individual cells, revealing distinct cell types involved, and their functional profile in better aiding the understanding of BBR’s role in modulating immune responses. Additionally, BCL6 binding to the STAT6 site of the IL-4 promoter suppresses the induction of immunoglobulin germline transcript [35]. Furthermore, it has been shown that BCL6 deficient mice have enhanced IgE class switching, whereas BCL6 and STAT6 double knockout mice do not produce any IgE [6]. To determine if BBR has any effect on the expression of BCL6, we performed gene expression analysis which showed a significant increase in expression of BCL6 with BBR treatment following anti-CD40/IL4 stimulation. We further validated these results by performing a ChIP assay to study the binding affinity of BCL6 and STAT6 on IgEh. Our results showed stronger binding of BCL6 and reduced binding of STAT6 in the IgEh promoter. Further studies using more robust binding assays, such as luciferase reporter assays and histone modifications, should be considered in future studies to understand BBR’s activity on IgE promoters.

For the first time, we investigated the anti-IgE effect of BBR in suppressing IgE synthesis by human tonsil cells. Novel natural compound-based anti-IgE therapies hold significant promise in the treatment and advancement of IgE-mediated allergic diseases. These small molecule compounds are potent, effective, safe and show broader application. Future strategies should focus on combining natural and biological therapies for enhanced anti-IgE response. These findings suggest the clinical relevance of these small molecule compounds, offering safer holistic alternatives for patients with IgE-mediated allergic diseases. Our results suggest that the overall IgE suppressive effect of BBR is due to multiple different regulatory mechanisms.

In conclusion, we, for the first time, demonstrated that BBR effectively suppresses IgE production in human tonsil cells from children undergoing tonsillectomy. BBR inhibited IgE production without any change in IgG levels and showed no association cytotoxicity following stimulation with anti-CD40/IL4 alone or in combination with TLR agonists poly I:C, Pam3CSK4, or both. This study further identifies key mechanisms associated with IgE inhibition. First, we showed the inhibition of IgE is associated with inhibition of STAT6 and enhancement of BCL6 binding in the IgE promoter region, both of which are critical for restricting class switch recombination to IgE. Second, we showed significant downregulation of NFκB1 expression, which partially was associated with enhanced expression of its inhibitor, NFκB1A. Further, in vivo studies are underway to observe BBR’s anti-IgE effects.

## 5. Conclusions

Overall, these findings further strengthen the anti-IgE effect of BBR and pave a path for future in vivo studies to support BBR as a potential therapeutic treatment for IgE-mediated allergic diseases such as food allergy.

## Figures and Tables

**Table 1:** Patient demographics.

ID	Age	Gender	Diagnosis
1	5	M	Obstructive Sleep Apnea
2	9	F	Enlarged tonsil
3	4	F	Tonsil hypertrophy
4	8	M	Tonsillitis
5	8	F	Enlarged tonsil
6	6	F	Enlarged tonsil
7	5	F	Obstructive Sleep Apnea
8	7	M	Tonsillitis
9	2	M	Obstructive Sleep Apnea
10	5	M	Enlarged tonsils

## Data Availability

The data that support the findings of this study are available from the corresponding author upon reasonable request.

## Supplementary Materials

The following supporting information can be downloaded at: https://www.mdpi.com/article/10.3390/cells14080591/s1, Figure S1. HPLC chromatogram and the chemical structure of berberine. Table S1. Primer sequence.
