# Supplementary material for: Berberine Suppression of Human IgE but Not IgG Production via Inhibition of STAT6 Binding Activity at IgE Promoter by BCL6
Source: Cells. 2025 Apr 14;14(8):591. doi: 10.3390/cells14080591 (PMC12026231; doi:10.3390/cells14080591)
Supplement: Supplementary file 1 [file cells-14-00591-s001.zip › supplemental figures.pptx]

## Slide 1
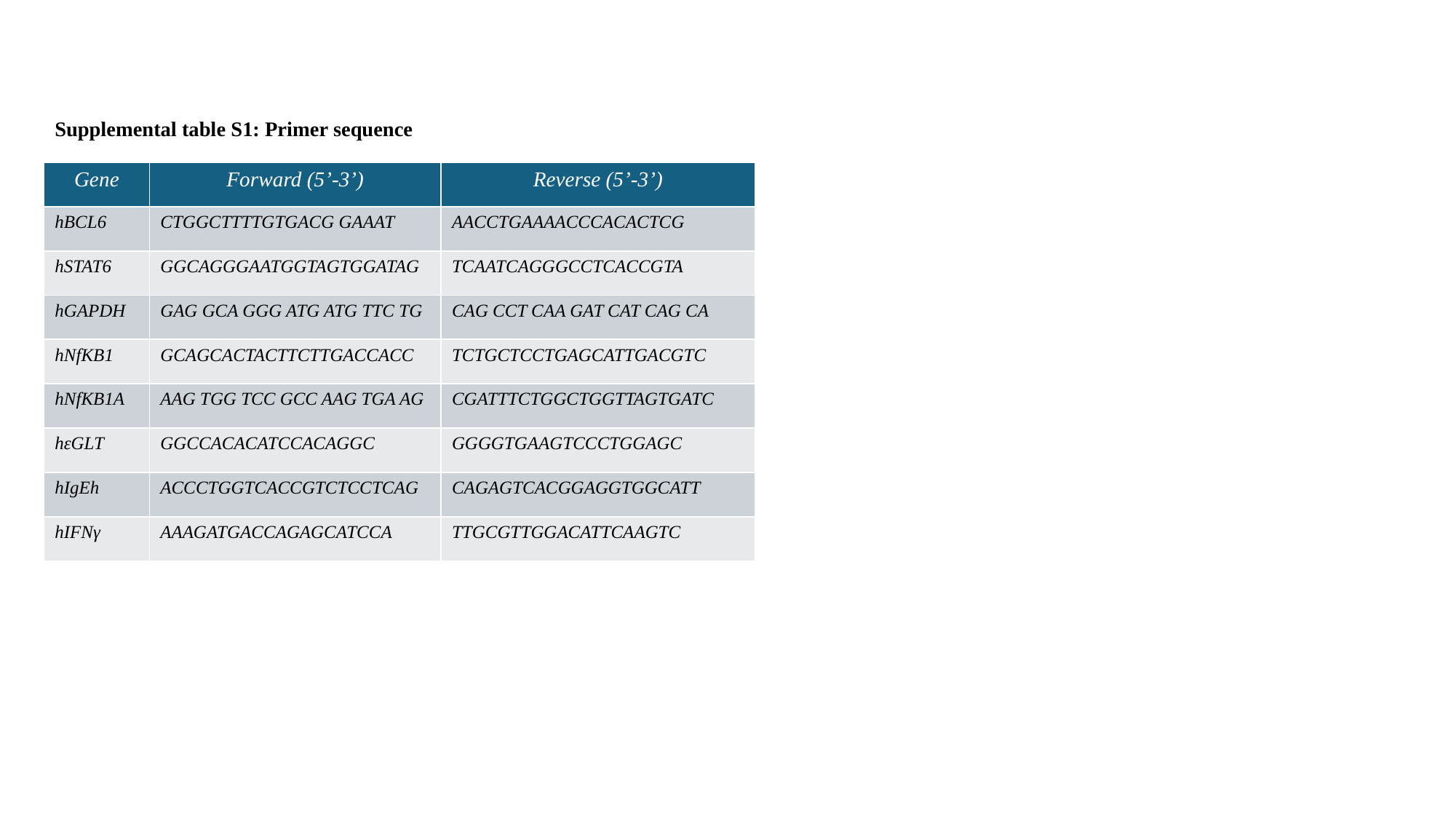

Supplemental table S1: Primer sequence
| Gene | Forward (5’-3’) | Reverse (5’-3’) |
| --- | --- | --- |
| hBCL6 | CTGGCTTTTGTGACG GAAAT | AACCTGAAAACCCACACTCG |
| hSTAT6 | GGCAGGGAATGGTAGTGGATAG | TCAATCAGGGCCTCACCGTA |
| hGAPDH | GAG GCA GGG ATG ATG TTC TG | CAG CCT CAA GAT CAT CAG CA |
| hNfKB1 | GCAGCACTACTTCTTGACCACC | TCTGCTCCTGAGCATTGACGTC |
| hNfKB1A | AAG TGG TCC GCC AAG TGA AG | CGATTTCTGGCTGGTTAGTGATC |
| hεGLT | GGCCACACATCCACAGGC | GGGGTGAAGTCCCTGGAGC |
| hIgEh | ACCCTGGTCACCGTCTCCTCAG | CAGAGTCACGGAGGTGGCATT |
| hIFNγ | AAAGATGACCAGAGCATCCA | TTGCGTTGGACATTCAAGTC |

## Slide 2
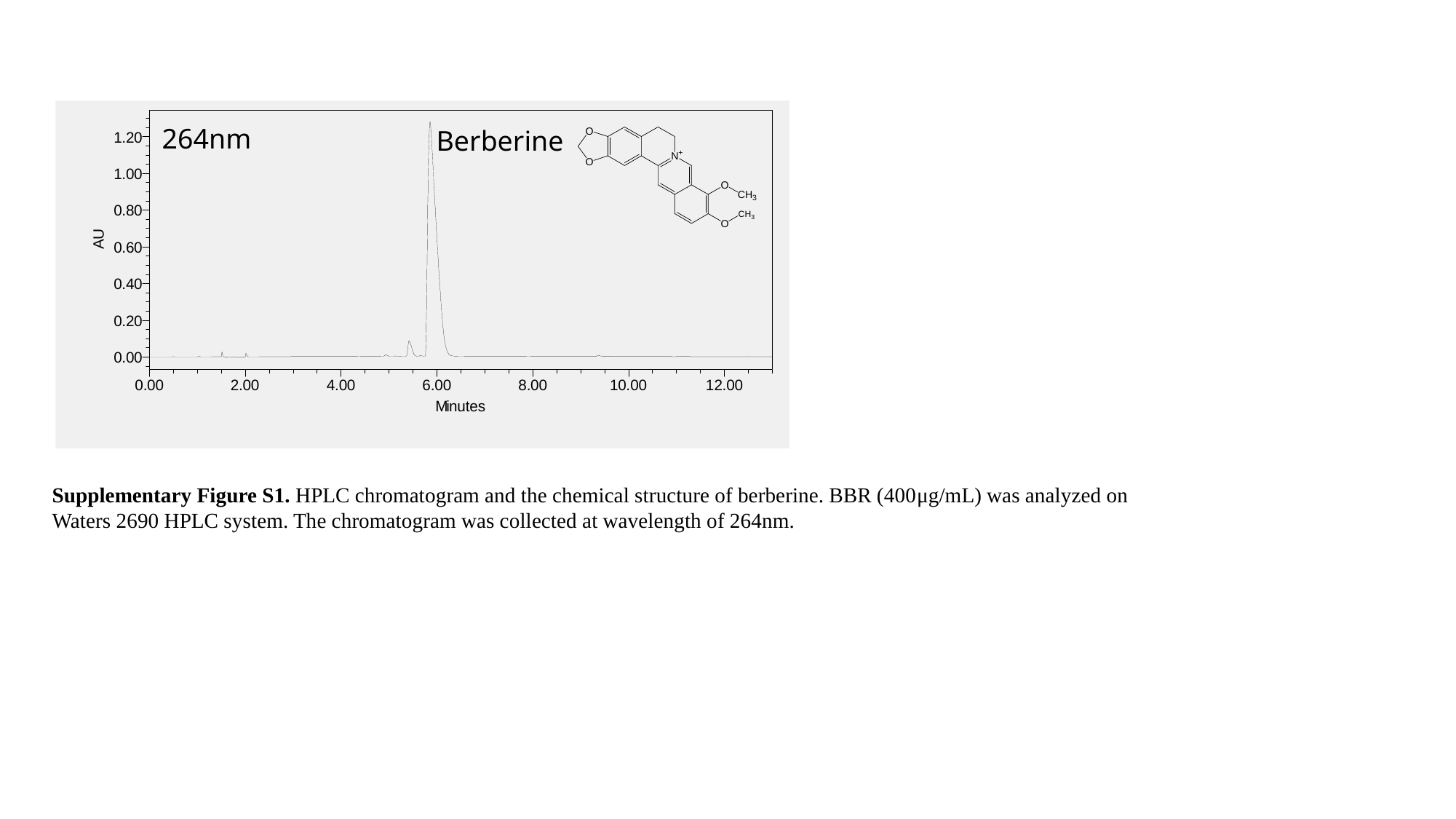

264nm
Berberine
Supplementary Figure S1. HPLC chromatogram and the chemical structure of berberine. BBR (400μg/mL) was analyzed on Waters 2690 HPLC system. The chromatogram was collected at wavelength of 264nm.
